# The Guidewire-assisted Drainage Catheter Placement in Chronic Subdural Hematoma

**DOI:** 10.3389/fsurg.2022.891119

**Published:** 2022-05-26

**Authors:** Bingjie Zheng, Chunlei Wang, Jinbiao Yao, Shiyi Zhu, Svetlana Meshcheryakova, Huaizhang Shi

**Affiliations:** ^1^ Department of Neurosurgery, The First Affiliated Hospital of Harbin Medical University, Harbin, China; ^2^ Bashkir State Medical University, Republic of Bashkortostan, Ufa, Russia

**Keywords:** guidewire-assisted technique, chronic subdural hematoma, subdural drain, iatrogenic complications, intracranial hemorrhages

## Abstract

**Background:**

Chronic subdural hematoma (cSDH) is a common neurosurgical pathology associated with older age. The burr hole drainage is a predominant technique with a lower incidence of recurrence and morbidity. The blind placement of the subdural drain could result in intracerebral hemorrhage. This paper describes a simple and reliable technique for drainage catheter placement in cSDH to reduce intracerebral hemorrhage.

**Methods:**

Forty-nine consecutive patients with cSDH were treated with The Guidewire-assisted Drainage Catheter Placement Technique between July 2019 and June 2021. Epidemiological, clinical and radiographical data were collected and reviewed. The operative technique consists of an angular guidewire tip and catheter. Under the navigation of the guidewire, the catheter is inserted into the subdural space and the length of catheter remaining in the subdural space was 4–5 cm. The catheter was tunneled subcutaneously and fixed at the point where it emerged from the scalp.

**Results:**

Forty-nine consecutive patients underwent 55 The Guidewire-assisted Drainage Catheter Placement. The gender distribution was 37 men and 12 women. The mean age was 69.3 years. The patients presented with headache (31 patients), weakness of limbs (28 patients), speech disturbances (7 patients), and Altered behavior (6 patients). Neither intracerebral hemorrhages nor post-operative seizure occurred. Forty-seven patients were improved after the operation. The recurrence occurred in one patient.

**Conclusions:**

The Guidewire-assisted Drainage Catheter Placement Technique is a reliable method for the insertion of a subdural catheter to evacuate of the Chronic Subdural Hematoma, and is associated with an extremely low risk to cortical structures and cerebral veins.

## Introduction

Chronic subdural hematoma (cSDH) is common in neurosurgical practice. The annual incidence of cSDH, at 5 per 100,000 in the general population, can increase to 58 per 100,000 in older age groups (>70 years) (1). There has been a steady, increasing incidence of cSDH as a result of prolonged life expectancy in developing countries in recent years (2).

The conventional therapeutic options for the treatment of cSDH include medical and surgical methods. For symptomatic patients with focal neurological deficit, surgical drainage is regarded as the treatment of choice. Three techniques have been described: twist-drill craniostomy, burr-hole craniostomy, and craniotomy. Recent articles state that burr hole drainage is a superior technique when compared to twist-drill craniostomy and craniotomy, due to a lower incidence of recurrence and morbidity (3–5). Therefore, burr-hole craniostomy with drainage has been popularized around the world. However, a seizure and intracerebral hemorrhage may occur peri-operation, which is mainly induced by a traumatic placement of the subdural drain. Pavlov et al. described a serious intracerebral hemorrhage which was induced by an intracranial cathter (6). Schoedel et al. found that the incidence of procedure-related complications, such as acute rebleed, intracranial bleeding and drainage mispositioning, was 3.9% (7). Hassler et al. reported that 77 complications, believed to be related to the surgical intervention, were observed in 376 patients (8). Therefore, avoiding these procedure-related complications is important for the reduction of mortality and morbidity in cSDH.

The present paper describes a guidewire-assisted technique which may minimize the complication risk of injury to cortical structures and cerebral veins, by using a guiding wire.

## Methods

In this retrospective study, we reviewed the data of 49 patients who underwent the guidewire-assisted drainage catheter placement for cSDH from July 2019 to June 2021 at Department of Neurosurgery, The First Affiliated Hospital of Harbin Medical University. This study was approved by the Harbin Medical University ethic committee. Epidemiological, clinical and radiographical data were collected and reviewed. All patient agreed to publication of clinical details and images.

### Surgical Technique

**Figure 1:**
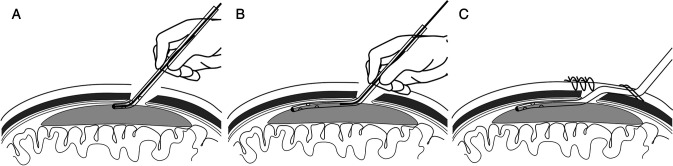
Step-by-step procedure for the guidewire-assisted technique. (**A**) The catheter (14F) with the guidewire at an angle (about 135 degrees) was inserted into the bone hole. (**B**) With the wire navigation, the 4–5 cm catheter was slowly pushed into the subdural space toward the frontal direction. (**C**) The guidewire was slowly withdrawn. The catheter was tunneled subcutaneously and fixed properly.

The operation was performed under monitored anesthesia care. The patient was placed typically in the lateral position with the affected side up, and the head elevated at 15 degrees. This position could avoid or reduce the incidence of pneumocephalus. A skin linear incision of approximately 3 cm and single burr-hole was made above the superior temporal line, generally near the parietal bulge. Dura mater and the outer membrane of the subdural hematoma was opened and coagulated by bipolar coagulation. The catheter (14F) with the guidewire at an angle (about 135 degrees) was inserted into the bone hole. The length was about 1 cm in the subdural space. This technique ensures the catheter was placed in the hematoma cavity and closed to the inner surface of the bone due to the angle of the tip. Then, with the wire navigation, the catheter was slowly pushed into the subdural space toward a frontal direction. The catheter length remained in the subdural space was 4–5 cm. Then the wire was slowly withdrawn. The catheter was tunneled subcutaneously and fixed at the point where it emerged from the scalp (Figure 1). Continuous irrigation was performed with a sterile saline solution at 37°C until the effluent was clear. The subdural space was filled with saline before closing the skin incision to minimize intracranial air collection. The subdural catheter was connected to a drainage system using a sterile technique.

### Statistical Analysis

Means, standard deviations, and medians were reported for continuous variables, and percentages were reported for categorical variables. Continuous variables were analyzed with a t-test, categorical variables were analyzed with a Chi-square test, statistical significance was defined as *p* < 0.05. Statistical analysis was performed using the statistical software SPSS (version 25, IBM Corp.).

## Results

### Demographics, Comorbidities, and Presentation

Forty-nine consecutive patients underwent 55 The Guidewire-assisted Drainage Catheter Placement. There were 6 bilateral and 43 unilateral hematomas. The mean age was 69.3 years.The gender distribution was 37 men and 12 women. The comorbidities included hypertension (34.7%), diabetes mellitus (14.3%) and coronary artery disease (12.2%). The most common presenting symptom was headache which was present in 65.3% of the patients. Other presenting symptoms included weakness of limbs in 28(57.1%), speech disturbances in 7(14.3%), and Altered behavior in 6(12.2%) the patients (Table 1).

**Table 1:** Summary of demographics, comorbidities, and presentation (*N* = 49 patients).

Parameter	Value
Age (Mean, range)	69.3 years, (39–87)
Male (*N*, %)	37 (75.5%)
Hypertension	17 (34.7%)
Diabetes mellitus	7 (14.3%)
Coronary artery disease	6 (12.2%)
Presentation (*N*, %)	
Headache	31 (65.3%)
Weakness of limbs	28 (57.1%)
Speech disturbances	7 (14.3%)
Altered behavior	6 (12.2%)

### Radiological, Clinical Outcomes and Copmplication

Preoperative and Postoperative measurements of hematoma thickness were displayed in Table 2. The maximal thickness of hematoma before operation ranged from 1.61–3.61 cm (2.31 ± 0.43 cm). After the evacuation, SDH maximal thickness was decreased to 0.21–2.31 cm (1.08 ± 0.46 cm)(*P* = 0.000,). The neurological status at admission was compared with that at the day of hospital discharge. The mean mRS at admission was 2.2. It was 1.0 at discharge (*P* = 0.000,). The neurological status of 45 patients (91.8%) improved after the operation. Neither intracerebral hemorrhages nor post-operative seizure occurred. The recurrence occurred in three patients (6.1%) at 3 months after surgery (Table 2).

**Table 2:** Result of radiological and clinical outcomes (*N* = 49 patients).

	Preop	Postop	*P*
The maximal thickness of hematoma (cm)	2.31 ± 0.43	1.08 ± 0.46	0.000
The mean mRS	2.2	1.0	0.000

*Preop, preoperative; Postop, postoperative.*

### Case Illustration

**Figure 2:**
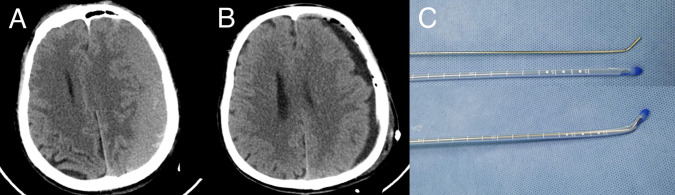
Case illustration. (**A**) The initial CT scan on presentation reveals the left chronic subdural hematoma. (**B**) CT of the head following the placement of drainage demonstrates the almost complete resolution of the left chronic subdural hematoma. (**C**) The guidewire and catheter used in the technique; the guidewire was bent at an angle of 135 degrees.

A 54-year-old male was referred to our department with a history of progressive right limbs weakness. He had been involved in a motor vehicle accident approximately one month prior. Physical examination revealed hemiparesis of the right arm and leg. The CT scan showed a large, chronic subdural hematomas on the left (Figure. 2A). We used the guidewire-assisted drainage catheter placement in this patient. Post-operatively, the patient’s hemiparesis improved rapidly. CT scan one day later revealed the subdural hematomas was disappeared nearly (Figure 2B). Figure 2C showed the guidewire and catheter used in the operation, the guidewire was bent at an angle of 135 degrees.

## Discussion

We present a retrospective study of 49 patients with a chronic subdural hematoma consecutively treated with a technique that we have not found reported previously. The guidewire-assisted technique may minimize the complication risk of injury to cortical structures and cerebral veins, by using a guiding wire. It probably could prevent postoperative seizures and severe intracranial hemorrhages.

cSDH is a common neurological condition that usually affects the elderly. At present, burr-hole surgery with a subdural closed-drainage system is the most commonly chosen strategy (9). Level 1 evidence also suggests the placement of a closed subdural drainage system at the time of burr hole evacuation can reduce symptomatic recurrence (4). However, A seizure and intracerebral hemorrhage are the frequent complications which could be a result of blind placement of the subdural drainage system (6, 8). Levin häNi et al found that among patients with subdural catheter drainage, the probability of epilepsy was 3.2%, and the probability of brain tissue injury was 2.8% (10). Another study from Minna rauhala et al found that the probability of epilepsy was 4.8%, and the probability of intracranial hematoma was 1.7% (11). Either with twist-hole or burr-hole methods, the drainage catheter is usually inserted into the subdural space blindly. Thus, the catheter tip may be placed into the brain parenchyma or injure the vessels on the cerebral surface. In addition, the catheter direction is not under control during insertion, and therefore, it can lead to drainage malpositioning (4). In this study, we demonstrated our 49 patients experiences of guidewire-assisted technique which could not only guide drainage, but also prevent damage the brain and vessel. Our results showed no complications such as postoperative seizures and intracranial hemorrhages occurred.

Various techniques have been described to facilitate placement of the drainage system to avoid the complications mentioned above. Some authors used subperiosteal drainage to treat cSDH. When this was compared to subdural drainage, it was found that subperiosteal drainage produced lower intracerebral hematoma and overall mortality, the surgical infection rate was significantly lower. However, repeat operations were higher than when subdural drainage was used (10, 12–15). Fichtner et al. used a nelaton catheter guard for placement of the subdural drain, to reduce the risk of damaging relevant structures such as cortical tissue or bridging veins. However, manipulation of the nelaton catheter may be difficult and complex. The catheter needs to be introduced into a bone hole, and withdrawn from the other hole (16). Therefore, The best way to place a catheter could be to place it into the subdural space which should avoid injuring the brain parenchyma and vessels, and this method should be easy to perform. The guidewire-assisted technique was safe and effective in our series.

The benefits of our proposed technique include increased catheter control and reduced complications when compared to traditional methods. Our technique is similar to the Seldinger Technique used in endovascular practice (17). Under guidewire support and navigation, the movement of the tip of the catheter in the subdural space is controlled with greater accuracy. Our guidewire tip is bent and this additional curvature of the wire allows the catheter to be inserted into the subdural space parallel to the surface of the brain, keeping the draining holes in the hematoma. Due to the contact with the hematoma in the subdural space, it is more helpful to drain the hematoma (13). Furthermore, because of the navigation of the wires, the catheters can be successfully implanted into the correct place. In general, the tip of the catheter is toward the frontal direction, as a recent systemic review found, it can reduce the recurrence of subdural hematoma (9). We used the guidewire-assisted technique in forty-nine consecutive patients with chronic SDH. Neither intracerebral hemorrhages nor post-operative seizure occurred and the recurrence only occurred in three patients. The technique described here is a reliable method for the insertion of a subdural catheter and is associated with an extremely low risk to damage cortical structures and cerebral veins.

The length of the catheter in the subdural space is 4–5 cm. A longer length of catheter will possibly damage the cerebrum or vessels at the edge of the hematoma and can induce seizures and intracerebral hemorrhaging. If the length is smaller than 4 cm, because of the stiffness of the catheter, its tip may jump into the brain parenchyma when the hematoma is evacuated rapidly. In addition, the indwelling drainage tube needs to be pushed slowly to prevent damage brain tissue. Meanwhile, attention should be paid to prevent pneumocranium, so as to further prevent recurrence.

Endoscope-assisted evacuation of cSDH is an established, although not widely used, technique. Main advantages of the endoscope-assisted technique are identification of membranes and septations and insertion of a catheter under direct visual control. It results in better placement of catheter in cavity for irrigation and removal of clot and fluid. It was found to be effective for removal of CSDH especially in septate hematomas and multiloculated hematomas (18). However, there needs for special training and the extra equipment (19). Meanwhile, it could increase risk of damage to the cortical surface or membrane due to rigid endoscope or by the rigid suction cannula. The technique described in this paper does not need extra equipment. Furthermore, its simplicity of use, allows for a relatively short learning curve, as seen with junior neurosurgeons. Our technique is associated with an extremely low risk to damage cortical structures and cerebral veins.

The main limitation of this study is that this is a retrospective series. In addition, the mount of patients is low. this may be difficult to get a high level of evidence supporting the use of The Guidewire-assisted Drainage Catheter Placement Technique. Nevertheless, the results suggest that patients with cSDH can benefit from the technique

## Conclusions

The technique described here is a reliable method for the insertion of a subdural catheter and is associated with an extremely low risk of cortical structures and cerebral veins. Additionally, its simplicity of use, allows for a relatively short learning curve, as seen with junior neurosurgeons.

## Data Availability

The original contributions presented in the study are included in the article/supplementary material, further inquiries can be directed to the corresponding author/s.
